# Biological Effect of Green Synthesis of Silver Nanoparticles Derived from *Malva parviflora* Fruits

**DOI:** 10.3390/ijms26178135

**Published:** 2025-08-22

**Authors:** Suzan Abdullah Al-Audah, Azzah I. Alghamdi, Sumayah I. Alsanie, Ibtisam M. Ababutain, Essam Kotb, Amira H. Alabdalall, Sahar K. Aldosary, Nada F. AlAhmady, Salwa Alhamad, Amnah A. Alaudah, Munirah F. Aldayel, Arwa A. Aldakheel

**Affiliations:** 1 Department of Biology, College of Science, Imam Abdulrahman Bin Faisal University (IAU), P.O. Box 1982, Dammam 31441, Saudi Arabia; naalaudah@iau.edu.sa (S.A.A.-A.); azalghamdi@iau.edu.sa (A.I.A.); sualsanea@iau.edu.sa (S.I.A.); iababutain@iau.edu.sa (I.M.A.); ekghareeb@iau.edu.sa (E.K.); aalabdalall@iau.edu.sa (A.H.A.); skdosary@iau.edu.sa (S.K.A.); smalhamad@iau.edu.sa (S.A.); 2220040220@iau.edu.sa (A.A.A.); 2 Basic and Applied Scientific Research Center (BASRC), Imam Abdulrahman Bin Faisal University (IAU), P.O. Box 1982, Dammam 31441, Saudi Arabia; 3 Department of Biology, College of Science, King Faisal University (KFU), Al-Ahasa 31982, Saudi Arabia; maldayel@kfu.edu.sa; 4 Department of Physics, College of Science, Imam Abdulrahman Bin Faisal University (IAU), P.O. Box 1982, Dammam 31441, Saudi Arabia; aldakheelarwa@gmail.com

**Keywords:** antibiotic-resistant bacteria, *Escherichia coli*, *Staphylococcus aureus*, *Malva parviflora* fruits, antibacterial, nanoparticles, antioxidant, biofilm

## Abstract

The search for novel natural resources, such as extracts from algae and plant for use as reductants and capping agents for the synthesis of nanoparticles, may be appealing to medicine and nanotechnology. This study aimed to use *Malva parviflora* fruit extract as a novel source for the green synthesis of silver nanoparticles (AgNPs) and to evaluate their characterization. The results of biosynthesized AgNP characterization using multiple techniques, such as UV–Vis spectroscopy, scanning electron microscopy (SEM), FTIR analysis, and zeta potential (ZP), demonstrated that *M. parviflora* AgNPs exhibit a peak at 477 nm; possess needle-like and nanorod morphology with diameters ranging from 156.08 to 258.41 nm; contain –OH, C=O, C-C stretching from phenyl groups, and carbohydrates, pyranoid ring, and amide functional groups; and have a zeta potential of −21.2 mV. Moreover, the antibacterial activity of the *M. parviflora* AgNPs was assessed against two multidrug-resistant strains, including *Staphylococcus aureus* MRSA and *Escherichia coli* ESBL, with inhibition zones of 20.33 ± 0.88 mm and 13.33 ± 0.33 mm, respectively. The minimum bactericidal concentration (MBC) was 1.56 µg/mL for both. SEM revealed structural damage to the treated bacterial cells, and RAPD-PCR confirmed these genetic alterations. Additionally, *M. parviflora* AgNPs showed antioxidant activity (IC_50_ = 0.68 mg/mL), 69% protein denaturation inhibition, and cytotoxic effects on MCF-7 breast cancer cells at concentrations above 100 µg/mL. These findings suggest that *M. parviflora*-based AgNPs are safe and effective for antimicrobial and biomedical applications, such as coatings for implanted medical devices, to prevent biofilm formation and facilitate drug delivery.

## 1. Introduction

Bacterial resistance to multiple antibiotics is a major global health issue. The 2022 Global Antimicrobial Resistance and Use Surveillance System (GLASS) report indicated an increase in the rate of resistance among some bacterial strains to antibiotics. For example, 42% of *E. coli* were reported to be resistant to third-generation cephalosporins [1]. This calls for a constant search for viable alternatives to these antibiotics. There has been considerable research recently on the use of silver nanoparticles in various applications, including in the medical and pharmaceutical fields, such as in cancer treatment formulations, drug delivery systems, and other medical applications [2,3,4,5,6,7], as well as in food [8], and agriculture [9,10]. Therefore, the use of nanoparticles is a potential alternative.

Several physical and chemical processes are employed to create NPs; however, these methods are not without their drawbacks, such as the need for extreme heat and pressure [11], and the toxicity of reducing and stabilizing agents [12]. Natural synthesis processes utilizing biological materials, like plants, are necessary for the production process of biomedical nanoparticles [13]. The term “green synthesis” describes the use of living entities, including bacteria, fungi, and plants, to produce NPs instead of harmful chemicals [14]. Biosynthesized nanoparticles are more stable and less toxic than chemically produced ones. The bioactive components of extracts (like plant extract), such as vitamins, trace metal ions, carotenoids, alkaloids, polyphenols, carbohydrates, fats, enzymes and proteins, are important substances that play important roles in synthesizing nanoparticles by serving as reducing agents, stabilizers, or building blocks.

Various studies have shown that natural extracts and microorganisms can be used to generate silver nanoparticles (AgNPs) that exhibit excellent antioxidant activity, which is superior to that of the original materials. This enhanced activity is believed to result from the components of the extracts adhering to the surface of the nanoparticles. Theoretically, this is attributed to the preferential sorption of extract components on the nanoparticle surface [15,16,17]. Additionally, anticancer activity of plant-biosynthesized AgNPs has been reported [18,19]. Furthermore, their anti-inflammatory effects have also been demonstrated [20].

*Malva parviflora* L. is an edible annual herbaceous plant native to Saudi Arabia; the Arabic common name is Khubaiza. According to Kew backbone distributions (KBDs), it is spread across many countries in Asia, Africa, South and North America, Europe, and Australia. It is a member of the *Malvaceae* family, which includes 249 accepted genera. *M. parviflora* is a decumbent herb with small flowers that develop after fertilization into schizocarp fruit containing multiple one-fruited mericarps. It is considered a medicinal plant because it contains various beneficial compounds. Glycosides, alkaloids, steroids, flavonoids, terpenes, saponins, tannins, and more are among its constituents. Its extracts demonstrate DPPH radical scavenging action, indicating both medicinal and culinary uses. Active ingredients, such as luteolin, cinnamic acid, pentadecanoic acid, and apigenin-7-glucoside, are also present in the leaves. *M. parviflora* exhibits a range of pharmacological activities, including antioxidant, anti-carcinogenic, anti-aging, anti-mutagenic, antibacterial, anti-irritant, antidiabetic, neuroprotective, antifungal, anti-ulcerogenic, hepatoprotective, analgesic, and anti-inflammatory properties [21]. It is used to treat bruises, sores, wounds, swelling, and broken limbs as a traditional remedy [22], as well as renal infections and kidney stones [23].

Several previous studies have investigated the possibility of biosynthesizing silver nanoparticles with *Malva* species. For example, green nanoparticles produced from *Malva sylvestris* flower extract were tested for antibacterial activity using disk diffusion on *Escherichia coli*, *Staphylococcus aureus*, and *Streptococcus pyogenes,* demonstrating effective antimicrobial properties [24]. *M. parviflora* leaf extract and biosynthesized silver nanoparticles inhibited the mycelial growth of fungal pathogens, including *Fusarium solani*, *Alternaria alternata*, *Fusarium oxysporum*, and *Helminthosporium rostratum*. Biosynthesized nanoparticles reduced the mycelial growth of *H. rostratum* the most, while the leaf extract was most effective against *F. solani* [25]. Additionally, a recent study demonstrated that the silver nanoparticles derived from *M. parviflora* L. could be an effective treatment option for laryngeal cancer [26].

Although the biosynthesis of *Malva parviflora* nanoparticles shows encouraging results, the literature on their anti-inflammatory and antioxidant activity is lacking. This study aims to evaluate the biological effects, including the antibacterial, antioxidant, anti-inflammatory and cytotoxic properties of Ag-nanoparticles synthesized for the first time from *M. parviflora* fruits, to assess their potential biomedical and pharmaceutical significance.

## 2. Results

### 2.1. Green Synthesis of Silver Nanoparticles

**Figure 1:**
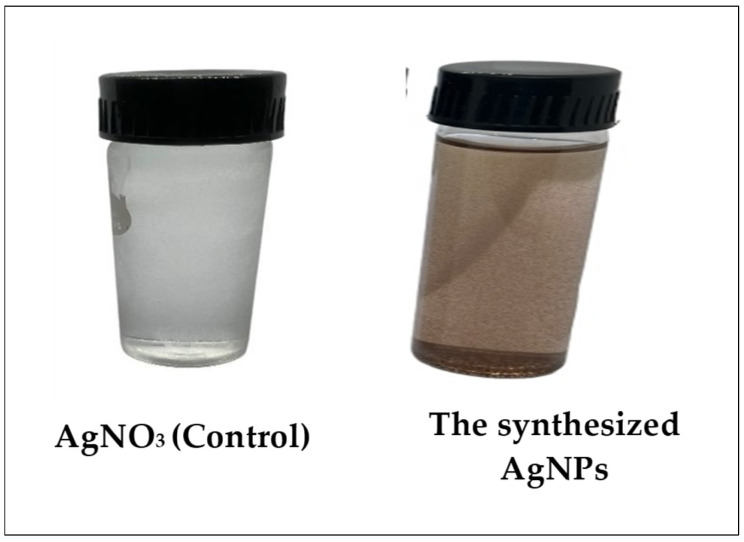
Green synthesis of silver nanoparticles with *M. parviflora* fruit extract.

AgNPs were biosynthesized from *M. parviflora* fruits. The ability of the plant extract’s active components to work as bioreductants for reducing silver ions to silver was investigated. After the addition of AgNO_3_, the color changed from pale yellow or from white to dark brown after 20 min (Figure 1). This color change indicates the production of AgNPs.

### 2.2. Characterization of AgNPs Biosynthesized from M. parviflora Fruits

#### 2.2.1. UV-Visible Spectral Analysis

**Figure 2:**
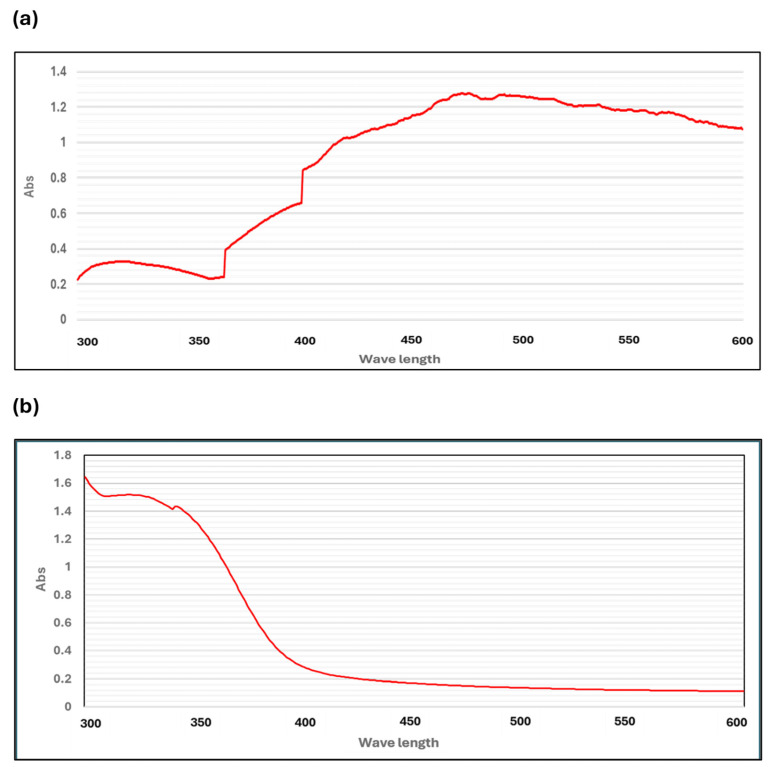
UV-Vis absorption spectrum. (**a**) *M. parviflora* plant nanoparticles; (**b**) *M. parviflora* extract.

The formation of *M. parviflora* AgNPs was determined by UV-visible spectroscopy, as shown in Figure 2a. The results reveal a maximum absorbance at 477 nm, which is linked to the AgNPs’ surface plasmon absorption. Additionally, a broad absorption band ranging from 400 to 520 nm was observed, which was not present in the UV-visible spectrum of the *M. parviflora* aquatica extract (Figure 2b). This difference indicates the successful synthesis of nanoparticles. 

#### 2.2.2. Technical Specifications of EDX

**Figure 3:**
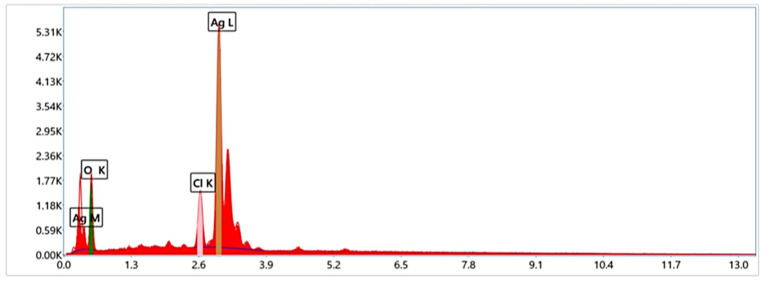
EDX spectrum of AgNPs synthesized with *M. parviflora* fruit extract.

EDX analysis revealed that AgNPs were biosynthesized, as indicated by a high signal in the silver area. Silver was present at 59%, followed by oxygen 35.3% and chlorine 5.54%, which was present at a smaller percentage (Figure 3).

#### 2.2.3. Zeta Potential Determination and Particle Size Distribution

**Figure 4:**
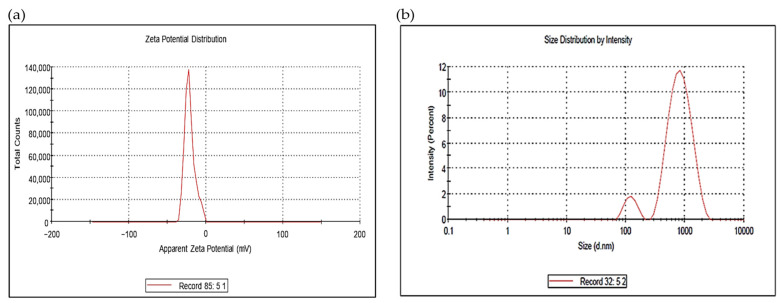
(**a**) Zeta potential determination and (**b**) particle size distribution.

Zeta potential was used to assess the *M. parviflora* AgNPs’ surface charge. (Figure 4a). The bio-synthesized AgNPs were stable, as they had a value of −21.2 mV. Figure 4b presents the hydrodynamic size distribution of the AgNPs based on DLS, which shows that the average size was 572 nm. The polydispersity index was determined to be 0.375.

#### 2.2.4. Scanning Electron Microscopy (SEM)

**Figure 5:**
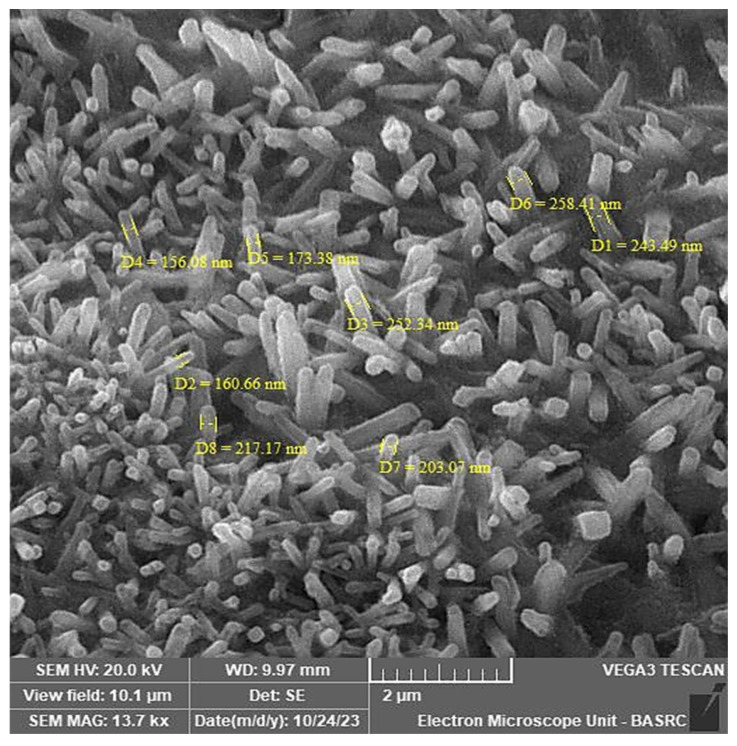
Scanning electron microscopy (SEM) image illustrating the typical rod-shaped morphology of silver nanoparticles (AgNPs) synthesized using *Malva parviflora* fruit extract. The image displays a distribution of nanorods, primarily in the nanometer size range. The scale bar represents 2 µm.

SEM analysis revealed that the *M. parviflora* AgNPs formed were well dispersed, with multiple needle-like and small cylindrical (nanorods) particle structures, and particle sizes of 156.08 to 258.41 nm (Figure 5).

#### 2.2.5. Fourier-Transform Infrared Spectroscopy (FTIR)

**Figure 6:**
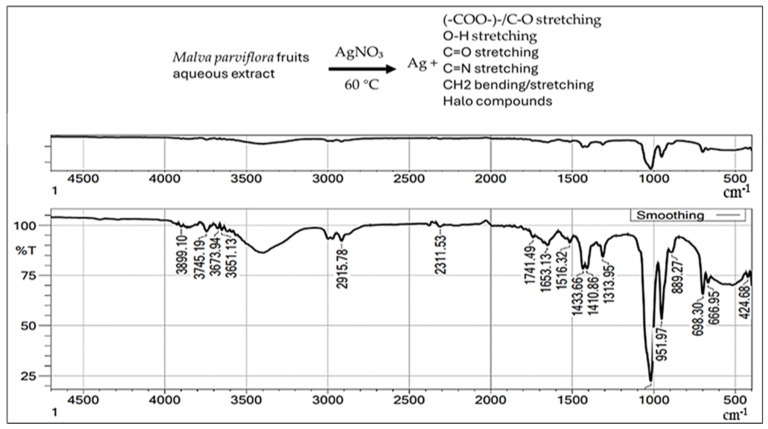
FTIR spectrum of AgNPs synthesized with *M. parviflora* fruit extract, along with a summarized list of functional groups.

The FTIR spectrum was employed in our study as a qualitative tool to confirm the functional groups involved in the biosynthesis of AgNPs. Figure 6 reveals various functional groups, including single, double, and fingerprint bonds.

The FTIR spectrum displays intense peaks at 3260.65, 2020.81, 1630.33, 1550.52, 1365.26, 1208.49, 1145.79, 991.88, 772.41, and 424.68 cm^−1^. The group’s stretching vibrations are shown, including polymeric hydroxyl compound O-H stretching, C-C stretching from phenyl groups, C=O stretching vibration, C-N stretching, lipids, COO symmetric stretching, CH2 bending/stretching vibrations C-O of mono-, 1112 oligo-, ester carbonyl (COOR), and carboxylate ion stretching (-COO-)-/C-O stretching vibration (amide), halo compounds, carbohydrates, and the pyranoid ring.

### 2.3. Evaluation of the Biological Activity of Biosynthesized AgNPs from M. parviflora Fruits

#### 2.3.1. Antibacterial Assay Against Multidrug-Resistant Bacteria

##### Diffusion Technique Assay

The findings of the antibacterial tests of *M. parviflora* AgNPs against *S. aureus* MRSA, *S. aureus* ATCC29213, *E. coli* ATCC25922, and *E. coli ESBL*, using the agar disc diffusion technique are shown in Table 1. The data reveal that *M. parviflora* AgNPs exhibited remarkable antibacterial activity against all bacterial strains compared to gentamycin (as a control treatment), with inhibition zones measuring 20.33 ± 0.88, 16.67 ± 0.33, 16.67 ± 1.33, and 13.33 ± 0.33 mm for *S. aureus* MRSA, *S. aureus* ATCC29213, *E. coli* ATCC25922, and *E. coli* ESBL, respectively. The highest effect was observed against *S. aureus* MRSA. 

##### Minimum Inhibitory Concentration (MIC) and Minimum Bactericidal Concentration (MBC)

The results of our investigation into the MIC and MBC are shown in Table 2. The MIC for all strains was 0.8 µg/mL, except for *S. aureus* ATCC29213, in which it was 3.125 µg/mL. The MBC was 1.56 µg/mL, except for *S. aureus* ATCC29213, in which it was 6.25 µg/mL.

##### Scanning Electron Microscope Analysis of the Antibacterial Effects of *M. parviflora* AgNPs on Bacterial Cells

**Figure 7:**
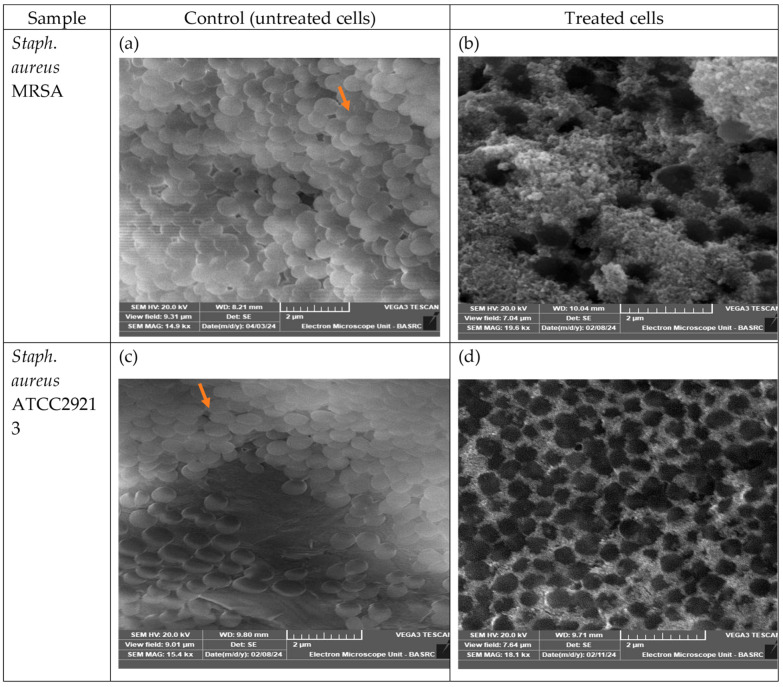
Scanning electron micrograph demonstrating the impact of *M. parviflora* AgNP treatment on *S. aureus* MRSA and *S. aureus* ATCC29213. cells. (**a**,**c**) Untreated cells exhibiting intact cellular structure and typical morphology (indicated by the orange arrow). (**b**,**d**) Both strains cells treated with nanoparticles, showing significant damage and perforation of the cell wall, which resulted in altered cellular structure and shape. Furthermore, most treated cells appeared to adhere together, indicating a disruption in biofilm formation compared to the untreated cells.

Among the tested bacterial strains, *S. aureus* (MRSA) exhibited the highest sensitivity to AgNPs synthesized from *Malva parviflora* fruits, followed by the standard *S. aureus* strain. Therefore, this strain was selected for ultrastructural analysis using scanning electron microscopy (SEM) to investigate the morphological changes induced by the nanoparticles. Examination of the SEM micrographs of untreated cells (Figure 7) revealed a higher number of cells with intact structures. After 2 h of culture with the AgNPs (100 µg/mL), SEM images showed that most *S. aureus* (MRSA) cells were damaged and perforated, which changed the cell structure and shape. Some cells appeared empty, and many were observed to be clumped together compared to untreated cells. *S. aureus* standard also showed changes in the cell structure and shape, with some cells appearing empty compared to untreated controls.

##### The Antibacterial Screening Effect of Biosynthesized *M. parviflora* AgNPs on Genomic DNA

**Figure 8:**
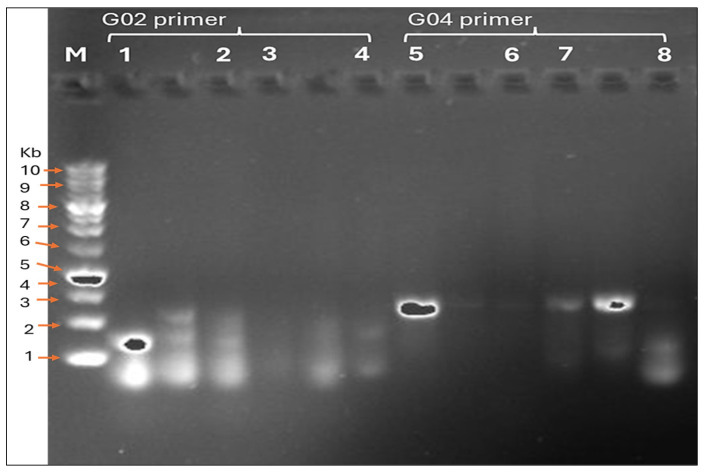
Gel electrophoresis analysis of RAPD-PCR amplification products of 2 primers (G02 and G04). The first well represents the 1 kb DNA marker (M), followed by the treatments. Wells 1, 2, 3, and 4 were amplified by G02 primer ((1) *S. aureus* MRSA not treated (control), (2) *S. aureus* MRSA treated by *M. parviflora* AgNPs, (3) *S. aureus* ATCC29213 not treated (control), (4) *S. aureus* ATCC29213 treated by *M. parviflora* AgNPs. Wells 5, 6, 7, and 8 were amplified by G04 primer ((5) *S. aureus* MRSA not treated (control), (6) *S. aureus* MRSA treated by *M. parviflora* AgNPs, (7) *S. aureus* ATCC29213 not treated (control), (8) *S. aureus* ST treated by *M. parviflora* AgNPs. The arrows on the left denote the molecular sizes determined from the 1 kilobase (Kb) DNA ladder analysis.

Based on the antibacterial screening results, *S. aureus* (MRSA) demonstrated the highest susceptibility to AgNPs synthesized from *Malva parviflora* fruits. Therefore, RAPD-PCR analysis was conducted with both *S. aureus* MRSA and *S. aureus* ATCC29213 to determine whether the nanoparticles exerted genetic effects on these strains. As shown in Table 3 and Figure 8, clear genotypic differences were observed between treated and untreated samples, with the *S. aureus* standard strain exhibiting up to 11 polymorphic bands generated by two selected primers after the AgNP treatment.

#### 2.3.2. Determination of Antioxidant Activity

**Figure 9:**
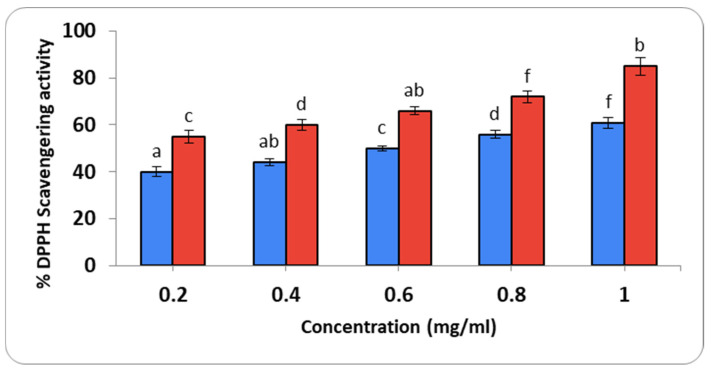
Estimation of antioxidant activities of *M. parviflora* AgNPs. Red is gallic acid and blue is *M. parviflora* AgNPs. The estimation of DPPH radical scavenging activity from different concentrations of *M. parviflora* AgNPs synthesized using *M. parviflora* leaf extract. The values with different subscript letters are significantly different according to ANOVA and Duncan’s multiple range tests (*p* < 0.05).

The DPPH radical scavenging activity for *M. parviflora AgNPs* at various concentrations (0.2–1.0 mg/mL) was analyzed using gallic acid as a standard (Figure 9). The color change from violet to yellow indicates the scavenging activity. Gallic acid displayed the greatest reducing action, with a scavenging efficiency of 61% at 1 mg/mL. The half-maximal inhibitory concentration (IC_50_) value was 0.68 mg/mL, while the IC_50_ value for gallic acid was 0.12 mg/mL.

#### 2.3.3. Anti-Inflammatory Action

##### Suppression of the Protein Denaturation

**Figure 10:**
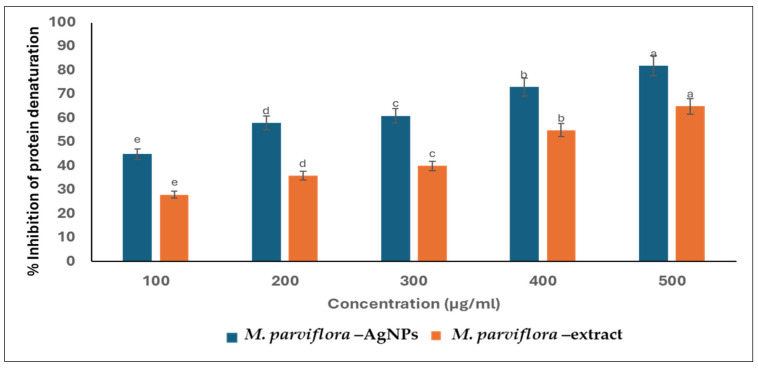
Estimation of antioxidant activities of Ag nanoparticles derived from *M. parviflora* fruits (*M. parviflora* AgNPs) using its extract as a control. The values with different subscript letters are significantly different according to ANOVA and Duncan’s multiple range tests. (*p* < 0.05).

The inhibition of protein denaturation is the chief mechanism of action for nonsteroidal anti-inflammatory drugs. Consequently, the capability of the plant and biosynthesized *M. parviflora* AgNPs to suppress the denaturation of protein was evaluated. Both the plant extract and biosynthesized *M. parviflora AgNPs* exhibited a significant anti-inflammatory action in a dose-dependent manner, and the lack of significant differences in this graph is intentional (Figure 10). At a concentration of 500 μg/mL, the percentage suppression of protein denaturation was 69% and 55% in the cases of *M. parviflora AgNPs* and the plant extract, respectively.

#### 2.3.4. Cytotoxicity Studies

**Figure 11:**
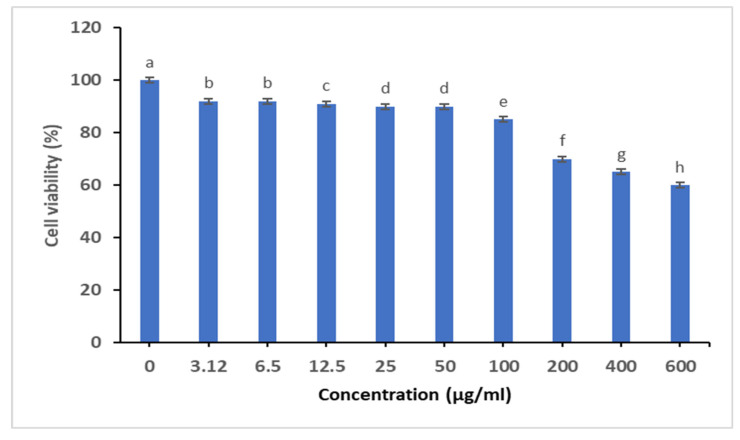
Cytotoxicity of *M. parviflora* AgNPs on the MCF-7 cell line. The dose-dependent effect of *M. parviflora* AgNPs was evaluated using an MTT assay after 24 h of treatment. The values with different subscript letters are significantly different according to ANOVA and Duncan’s multiple range tests. (*p* < 0.05).

The cell viability of *M. parviflora* AgNPs was observed using an MTT test on the MCF-7 cell line. As established in (Figure 11), *M. parviflora* AgNPs only exhibited cytotoxicity at concentrations above 100 µg/mL, and the lack of significant differences in this graph is intentional.

## 3. Discussion

Given the increasing rates of antibiotic resistance among human pathogenic bacteria, this study aimed to explore an environmentally friendly method for synthesizing silver nanoparticles (AgNPs) using *Malva parviflora* fruits and to evaluate their biological effects. Green synthesis presents a promising alternative with dual benefits for health and the environment.

The successful synthesis of silver nanoparticles (AgNPs) from *M. parviflora* under mild conditions was confirmed using multiple characterization techniques. Scanning electron microscopy (SEM) revealed the formation of needle-like, small, cylindrical, and regular nanoparticle structures. This morphology is uncommon in green synthesis methods, as most previous studies have reported spherical nanoparticles.

Previous studies have indicated that variation in nanoparticle shapes may be related to the unique biochemical composition of the plant extract used. Balan et al. (2016) reported that Lonicera japonica leaf extract produced spherical and hexagonal nanoparticles [27]. Similarly, Kajani et al. (2014) showed that ethanolic extract of *Taxus baccata* resulted in silver nanoparticles with hexagonal and truncated triangular shapes [28]. Furthermore, a review by Vanlalveni et al. (2021) concluded that environmental factors, such as the type of plant extract, temperature, silver nitrate concentration, and pH, significantly influence the size and shape of green-synthesized nanoparticles [29]. Therefore, further studies will be required to investigate the influence of different physical conditions on *M. parviflora* AgNPs.

EDX analysis verified the particles’ elemental makeup, with silver comprising 59%, followed by oxygen (35.3%) and chlorine (5.54%), in agreement with previous findings [25]. In addition, Kapoor et al.(2022) reported that EDX analysis indicated the presence of carbon, oxygen, and nitrogen, which could be attributed to the phytoconstituents of RIWS (*Rhodiola imbricata* and *Withania somnifera*) root extract, which are located on the surface of AgNPs [30]. And Farooqi et al. (2024) mentioned that synthesized *Cicer arietinum* (CA) AgNPs contained carbon (C), oxygen (O), and silver (Ag), according to EDS analysis [31]. The highest Ag peak confirms the successful synthesis of silver nanoparticles using *M. parviflora* extract. The presence of oxygen and chlorine peaks suggests that certain phytochemicals from the plant extract, containing functional groups such as C–O and C–Cl, were bound to the nanoparticle surfaces. These findings, supported by both EDX and FTIR analyses, provide a comprehensive understanding of the structural and chemical characteristics of *M. parviflora* AgNPs.

FTIR analysis indicated the presence of functional groups that likely play roles in the reduction and stabilization of AgNPs. The observed peaks at 3260.65, 2020.81, 1630.33, and other wavelengths correspond to hydroxyl, alkyl, carbonyl, lipids, ester carbonyl, amide, halo compounds, and phenyl-related vibrations, consistent with earlier studies [25,32]. These functional groups suggest the involvement of plant secondary metabolites in nanoparticle formation.

UV–Vis spectroscopy confirmed the presence of surface plasmon resonance (SPR), with absorption peaks at 477 nm, further supporting AgNP formation. Banu et al. (2021) noted that the particle size, shape, and the medium in which the AgNPs are suspended all play a role in the specific absorption band that these particles display, which is typically in the visible spectrum and can range from 400 to 500 nanometers [33]. In addition, Yusuf-Salihu et al. (2025) mentioned that the absorption peak indicates that the nanoparticles were dispersed evenly throughout the solution, suggesting that the silver ions (Ag+) were reduced to metallic silver (Ag0) [34]. In this study, a wide absorption band ranging from 400 to 520 nm was detected. This absorption band widening has been previously documented in green-synthesized AgNPs, where the size, shape, and aggregation state of the nanoparticles are affected by the complexity of the reducing and stabilizing agents in plant extracts [35]. Based on our results, we suggest that the phytochemicals in the biosynthesized AgNPs from *M. parviflora* fruits impacted the growth and nucleation of nanoparticles by acting as reducing and capping agents. This led to a size distribution and geometric shapes, which were verified by SEM analysis.

The nanoparticles showed a zeta potential of −21.2 mV, indicating good colloidal stability. This aligns with the results reported by Osman et al. and Hassan et al. [36,37], who also observed stable silver nanoparticles with negative surface charges [31,38]. In addition to enhancing colloidal stability, surface charge can influence the biological activity of AgNPs, particularly their interaction with bacterial membranes. Li et al. (2010) demonstrated that silver nanoparticles can compromise membrane integrity, leading to leakage of proteins and reducing sugars, as well as the inactivation of respiratory chain enzymes in *E. coli*, ultimately resulting in bacterial cell death [39]. These findings suggest that AgNPs may exert their antibacterial effects through a combination of membrane disruption and biochemical interference, beyond electrostatic interactions.

The silver nanoparticles synthesized from *M. parviflora* exhibited notable antibacterial activity against the tested strains, with the highest inhibition zone recorded against *S. aureus* MRSA (20.33 ± 0.88 mm). These findings highlight the broad-spectrum efficacy of AgNPs synthesized via green methods, capable of targeting both G+ve and G-ve bacteria. These findings align with the study by Madisha, (2024), which demonstrated the antimicrobial potential of *Malva parviflora* extracts as a promising natural alternative to conventional antibiotics [40]. The MIC ranged from 0.8 µg/mL to 3.125 µg/mL, while the MBC ranged from 1.56 µg/mL to 6.25 µg/mL. These values indicate a potent antibacterial effect, consistent with earlier research [25,36]. The results indicated that *S. aureus* MRSA exhibited higher sensitivity than *S. aureus* ATCC29213. While MRSA is generally known to be more challenging to treat [41], its traditional antibiotic resistance mechanisms may not apply to AgNPs. MRSA’s resistance to beta-lactam antibiotics relies on the production of the PBP2a protein, which reduces binding affinity for these antibiotics. However, this specific mechanism does not influence the mode of action of silver nanoparticles [42].

SEM images revealed morphological damage caused by *M. parviflora* AgNPs under the current experiment conditions. Treated *S. aureus* cells exhibited disrupted membranes, cytoplasmic leakage, and aggregation. These effects confirm the physical damage induced by AgNPs on bacterial structures, as reported in similar studies [43,44]. AgNPs interact with the bacterial cell wall, particularly the lipopolysaccharide layer, by binding to sulfhydryl groups (–SH) in structural proteins or enzymes within the wall. This interaction leads to pore formation, a subsequent decrease in intracellular turgor pressure due to the leakage of water and ions, and ultimately, cellular structural collapse. This is visible as cell shrinkage or complete collapse, as shown in Figure 7b,d.

RAPD-PCR was used to assess possible genetic alterations in *S. aureus* MRSA and *S. aureus* ATCC29213 following treatment with AgNPs. The results show clear polymorphic patterns in treated cells compared to controls. These genotypic changes suggest that AgNPs can interfere with bacterial DNA, in line with the results by Khasapane et al., Salama et al., and Altemimi et al. [45,46,47]. There are several proposed mechanisms to explain these findings based on previous studies. One possible explanation is that the shape of the silver nanoparticles synthesized from *M. parviflora* fruits (rod and needle-like shape) may play a significant role. According to Alshareef et al., truncated octahedral AgNPs exhibited more activity than spherical ones, possibly because of variations in active facets, surface energies, and surface area [48]. In addition, metal nanoparticles exhibit a preference for electrostatic interactions with bacterial lipopolysaccharide and biofilm matrix polysaccharides due to their negatively charged nature [49]. AgNPs can also enhance antibacterial efficacy by penetrating bacterial membranes and releasing Ag+ ions directly into the cytoplasm [50]. The interaction between DNA and ions of gold and silver caused DNA damage. This finding led to the conclusion that biofilm inhibition and cell wall disruption were caused by the damaging interactions between gold and silver ions with the DNA [48,49,50].

In terms of antioxidant activity, the DPPH assay revealed an IC50 value of 0.68 mg/mL, indicating substantial free radical scavenging potential. This result aligns with previous reports highlighting the antioxidant properties of biosynthesized AgNPs and the role of polyphenols from plant extracts in enhancing these effects [51,52,53]. Given the overall phenolic concentration of the *Malva* sp. extract, it exhibits high free radical scavenging activity [22].

Anti-inflammatory activity was assessed through the inhibition of protein denaturation, a known mechanism underlying inflammation. AgNPs synthesized from *M. parviflora* demonstrated notable inhibition, which may be attributed to the bioactive metabolites capping the nanoparticles. Similar anti-inflammatory effects have been observed in AgNPs synthesized using other plant extracts, such as Curcumin and *Asparagus racemosus* [54,55]. Rhimi et al. found that *M. parviflora* fruits contained high levels of primary metabolites, including proteins, soluble sugars, and starch, along with secondary metabolites, such as oil content, total flavonoids, and polyphenols [56]. Due to these contents, particularly the polyphenols, fruit extracts demonstrate significant antioxidant activity. Furthermore, antimicrobial, antiviral, and anti-inflammatory properties are found in antioxidant compounds [57,58].

Finally, cytotoxicity assays on the MCF-7 human breast cancer cell line indicated that AgNPs did not exhibit toxic effects at MIC concentrations. This suggests that the nanoparticles are biocompatible, supporting their potential for biomedical applications. These findings are consistent with previous studies that link AgNP safety to the nature of the plant-based reducing agents used in synthesis [59,60].

## 4. Materials and Methods

### 4.1. Source of the Plant Sample

**Figure 12:**
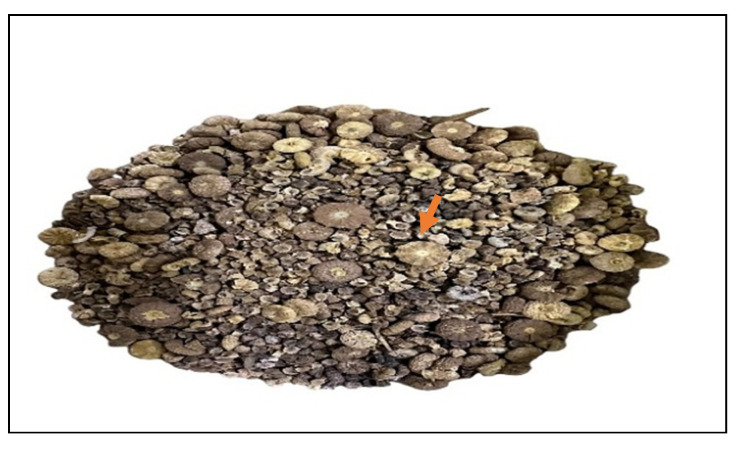
Fruits of *Malva parviflora*. A close-up view showcasing the distinctive schizocarpic fruits, commonly known as “cheesewheels” due to their segmented, disc-like appearance. Each fruit (indicated by the arrow) is composed of several mericarps (individual segments) arranged radially, typically exhibiting a dry, brown to grayish-brown coloration upon maturity.

Fruits of *Malva parviflora*, a wild medicinal plant shown in Figure 12, were obtained from a local seed vendor. 

### 4.2. Green Synthesis of Silver Nanoparticles (AgNPs)

The fruits of *Malva parviflora* were thoroughly washed with distilled water, dried, and then ground. Then, 10 g of the ground fruits was mixed with 100 mL of deionized distilled water, incubated in a water bath at 80 °C for 10 min, and then filtered through Whatman filter paper.

To generate silver nanoparticles, 10 mL (1 equiv.) of the aqueous fruits extract was mixed with 190 mL of 1 mM (19 equiv.) silver nitrate (AgNO_3_) solution. The mixture was placed in a water bath at 60 °C for thirty minutes (under mild conditions). The color change of the solution was monitored as a visual indicator, confirming the successful formation of silver nanoparticles.

The nanoparticle solution was stored in opaque, sealed containers at 4 °C for further use. Subsequently, 100 mL of the nanoparticle suspension was centrifuged at 4700 rpm for 20 min. The collected pellet was then dried in an oven at 40 °C for downstream applications [37].

### 4.3. Silver Nanoparticle Characterization 

#### 4.3.1. UV Spectral Analysis

The reduction of silver ions to silver nanoparticles was monitored using UV-Visible spectroscopy. A spectrophotometer (Shimadzu 8400, Kyoto, Japan) operating in the 200–600 nm wavelength range was utilized for the analysis. The *M. parviflora* AgNPs and the *M. parviflora* aqueous fruit extract (as a control) were both examined. A 2 mL solution was placed into a plastic cuvette, and the maximum absorbance wavelength was recorded to confirm nanoparticle formation [37].

#### 4.3.2. Technical Energy-Dispersive X-Ray Spectroscopy (EDX) Specifications

The EDX technique was used to determine the elemental makeup of the synthesized silver nanoparticles. The collected dried AgNPs synthesized from *M. parviflora* (previously described) were analyzed using a scanning electron microscope integrated with an EDX detector. (Tescan, Brno, Czech Republic). This setup enabled precise identification and quantification of the elements present in the sample, providing valuable insight into the composition and structural characteristics of the AgNPs across various applications [37].

#### 4.3.3. Zeta Potential Determination and Particle Size Distribution

Zeta potential and particle size distribution measurements were conducted using a spectrophotometer from Malvern Analytical, Enigma Business Park, UK. These measurements provided data on the surface charge, stability, hydrodynamic size, and polydispersity index (PDI) of the synthesized AgNPs.

To determine the hydrodynamic size and polydispersity index of the produced nanoparticles, we employed dynamic light scattering (DLS). Zeta potential is a critical indicator of colloidal stability in aqueous solutions [61].

#### 4.3.4. Scanning Electron Microscopy (SEM) 

Scanning electron microscopy (SEM) was employed to examine the shape and structural characteristics of the biosynthesized silver nanoparticles (AgNPs). The analysis was conducted using a Tescan VEGA3 SEM system (Tescan, Brno, Czech Republic), equipped with detectors for secondary electrons (SE), backscattered electrons (BSE), and low vacuum secondary electrons (LVST) [62].

The instrument featured a chamber with an internal diameter of 160 mm and a maximum specimen height capacity of 36/34 mm. This setup enabled high-resolution imaging to confirm the successful biosynthesis and assess the surface morphology of the AgNPs [63].

#### 4.3.5. Fourier-Transform Infrared Spectroscopy (FTIR)

This analysis was used to characterize the functional groups involved in the formation and stabilization of the biosynthesized silver nanoparticles [64]. The analysis was performed using an FTIR-8400 spectrometer (Shimadzu, Kyoto, Japan), with measurements recorded in the range of 500 to 4500 cm^−1^. This allowed identification of key chemical bonds and functional groups associated with the AgNPs [65].

### 4.4. Evaluation of the Biological Activity of Biosynthesized AgNPs from M. parviflora Fruits

#### 4.4.1. Antibacterial Assay Against Multidrug-Resistant Bacteria

##### Bacterial Isolates Source

The bacterial strains were two multidrug-resistant bacteria, as follows: *Staphylococcus aureus* MRSA (ID: PQ097594 in GenBank) and *Escherichia coli* ESBL (ID: PQ097597 in GenBank), both isolated from patients (patients’ consent was obtained). Additionally, two standard bacterial strains were used: *Escherichia coli* (ATCC25922) is a G-ve (Gram negative) bacteria, and the other is *Staphylococcus aureus* (ATCC29213) which is a G+ve (Gram positive) bacteria. All bacterial strains were obtained from King Fahd University Hospital laboratories, Eastern region, Saudi Arabia.

##### Diffusion Technique Assay

The antibacterial activity of the biosynthesized AgNPs from *M. parviflora* fruits was evaluated against the previous four bacterial strains using the agar well diffusion method, as described by Ferraro (1999) [66]. A total of 0.5 mL of bacterial culture (18–24 h old) standardized to 1.5 × 10^8^ CFU/mL (0.5 McFarland standard) was inoculated onto sterile Petri dishes. Subsequently, 15 mL of nutrient agar was poured into each plate.

Once the agar solidified, 5 mm wells were punched using a sterile cork borer. Then, each well was filled with 50 µL of AgNP solution. Gentamicin (GM 30 µg) served as the positive control, while AgNO_3_ (50 µL) was used as the negative control. The plates were refrigerated for 1 h to allow for diffusion of the substances, then incubated at 37 °C for 18–24 h. Antimicrobial activity was assessed by measuring the diameter of the inhibition zones around each well in millimeters. All experiments were conducted in triplicate [37].

##### Determination of Minimum Inhibitory Concentration (MIC)

The lowest antimicrobial concentration that visibly inhibited bacterial growth after overnight incubation was termed the MIC. The values of MIC were determined following the method described by Ababutain and Alghamdi [67].

##### Determination of Minimum Bactericidal Concentration (MBC)

The minimum bactericidal concentration (MBC) was determined using the pour plate method. The MIC experiment concentrations that did not show bacterial growth were individually transferred to Petri dishes. They were subsequently mixed well with nutrient broth medium, which had been liquefied and poured on top. After 24 h of incubation at 37 °C, the plates were checked. Plates were marked as MBC if they did not show any signs of bacterial colony growth. Each experiment was performed in triplicate [67].

##### Scanning Electron Microscope Analysis of the Antibacterial Effects of *M. parviflora* AgNPs on Bacterial Cells

SEM analysis was used to examine the morphological changes on the surface of the bacteria. Microbial cultures were grown in nutrient broth (NB). For this analysis, the more sensitive bacteria, either *S. aureus* or *E. coli*, in the diffusion technique assay were selected. Bacterial cultures were grown in nutrient broth (NB) overnight at 37 °C. This test included the control (NB medium + selected bacteria) and the treatments, which included the selected bacteria inoculated with a combination of 1 mL (1 equiv.) of NB media and 100 μL (0.1 equiv.) of biosynthesized AgNPs at a concentration of 4.26 nM. The tubes were incubated in an incubator shaker at 37 °C, set at 180 rpm for 2 h. The mixture was then centrifuged at 12,000 rpm for 10 min, and the resulting pellet was resuspended while the flow-through was discarded. In the next step, cells were fixed with 10% formaldehyde, dehydrated with serial methanol, and air-dried. These samples were then examined using a Tescan VEGA3 SEM (Tescan, Brno, Czech Republic) [68].

##### Screening Experiment on Biosynthesized AgNPs’ Antibacterial Effect on Genomic DNA

Random amplification of polymorphic DNA (RAPD) analysis was conducted to determine if the biosynthesized AgNPs from *M. parviflora* fruits influenced the bacterial genome. The more sensitive bacteria (*S. aureus* or *E. coli*) in the diffusion technique assay were chosen for this experiment. Bacterial strains were cultured in 1 mL of NB medium at 37 °C in 2 mL sterile microcentrifuge tubes and incubated overnight. After overnight incubation, two groups were established: the first one was treated with 100 μL (at a concentration of 4.26 nM) of biosynthesized AgNPs, while the second served as the control, untreated group. All samples were placed in an incubator shaker at 37 °C, set at 100 rpm for two hours. For DNA extraction, cells were harvested by centrifugation at 12,000 rpm for 3 min. The supernatant was discarded, and the cells were washed twice with 0.85% (*w*/*v*) NaCl before chromosomal DNA isolation, followed by another centrifugation at 12,000 rpm for three mins and discarding the supernatant. The cell pellet was then transferred to a sterile microcentrifuge tube. DNA was extracted using the Genomic Extraction Kit (Molecule ON, Auckland, New Zealand) following the manufacturer’s protocol, and the extracted DNA was stored at −16 °C for further use [47].

Two oligonucleotide primers were used, as shown in Table 4. All oligonucleotide primers were produced by Molequle-on, Auckland, New Zealand.

PCR amplification for RAPD reactions was performed in a 20 μL reaction mixture consisting of 11 μL PCR Master Mix (2×), 2 μL of each primer, 4 μL water, and 3 μL of the DNA template (Molequle-on, Auckland, New Zealand). Polymerase chain reaction was conducted using a Bio-Rad S1000™ Thermal Cycler (Hercules, CA, USA). PCR steps were programmed as follows:

The temperature profile was determined as follows: An initial denaturation step was carried out at 94 °C for 4 min, followed by annealing at 36 °C for 1 min, elongation at 72 °C for 5 min, and 40 cycles of denaturation and extension at 72 °C for 5 min. Finally, an extension at 4 °C for the remaining time was performed [69].

The amplified products were detected by gel electrophoresis. The RAPD-PCR samples (20 µL) were run in a 1% (*w*/*v*) agarose gel and stained with 5 μL of Visulala NA Nucleic Acid Stain in Tris-borate-EDTA buffer for one hour at 80 V and 400 mA, P120 w. The 1 kb DNA Marker (Molecule ON, Auckland, New Zealand) was used as a DNA size marker [47]. The results were visualized, photographed, and analyzed using the BIO-RAD Gel Doc^TM^ EZ Imager Hercules, CA, USA and Image Lab software. ver. 6.0.0.25 (Bio-Rad Laboratories, Hercules, CA, USA).

#### 4.4.2. Determination of Antioxidant Activity

Using the DPPH radical scavenging experiment, the AgNPs were utilized to quantify the antioxidant activity [70]. Using gallic acid as the standard, the decrease in DPPH was measured spectrophotometrically at 517 nm in comparison to the blank. The IC_50_ and percentage activity were assessed.

% activity = ((Ac − As)/Ac) × 100(1)

where Ac is the absorbance of the control, and As is the absorbance of the AgNPs mixture.

#### 4.4.3. Anti-Inflammatory Activity of the Biosynthesized AgNPs

##### Suppression of the Protein Denaturation

As previously mentioned by Souza et al. (2006) [71], the suppression of protein denaturation was utilized to assess the anti-inflammatory activity of the *M*. *parviflora* fruit extract and the biosynthesized silver nanoparticles. To 2.5 mL of PBS, 2 mL of *M*. *parviflora* extract was added, along with an egg solution (this was performed by adding 0.2 mL of fresh albumin (egg white) to 2.8 mL of phosphate buffer of PH 7.4). The mixtures were incubated at 37 °C before being heated to 70 °C. A spectrophotometer set at 660 nm was used to measure the turbidity. The percentage of suppressed protein denaturation was calculated using the following formula:

Percentage of suppression of protein denaturation = 100 × (1 − Absorbance (sample)/Absorbance (Control))(2)

#### 4.4.4. Cytotoxicity Study on Breast Cancer Cell Line

The MCF-7 breast cancer cell line was donated by the Leibniz Institute DSMZ German Collection of Microorganisms and Cell Cultures. Cells were cultured in Dulbecco’s Modified Eagle Medium (DMEM).

Using an MTT cell proliferation test kit to measure cell viability, the cytotoxicity of the AgNPs was determined. After seeding the cells on plates, they were incubated for 48 h with different concentrations of silver nanoparticles. The cells were then washed with PBS and cultured in a fresh medium containing MTT. The optical density was measured at 550 nm using an ELISA plate reader (Bio-Rad, Hercules, CA, USA). [72].

### 4.5. Statistical Analysis

SPSS 2007 (Ver. 17.0) was used for ANOVA calculations in order to investigate the ability of plant nanoparticles to inhibit selected bacterial strains. The significance value was measured at *p* < 0.01.

## 5. Conclusions

This study is the first report on the green-synthesized silver nanoparticles derived from *M. parviflora* fruits, revealing negative rod- and needle-like shaped nanoparticles with a size distribution at 572 nm by DLS analysis. In addition, the surface plasmon resonance (SPR) of the AgNPs exhibited a maximum absorbance peak at 477 nm, which was absent in the UV-visible spectrum of the aqueous extract of *M. parviflora*. This distinct difference confirms the successful synthesis of silver nanoparticles. FTIR spectroscopy identified functional groups, including hydroxyl, carbonyl, ester carbonyl, alkyl, amide, halo compounds, and phenyl-associated vibrations, indicating the presence of natural organic compounds from the plant extract that likely contributed to the reduction and stabilization of the nanoparticles. EDX analysis confirmed the elemental composition, revealing silver (59%), oxygen (35.3%), and chlorine (5.54%), further supporting the role of phytochemicals in nanoparticle synthesis. These biosynthesized AgNPs demonstrated strong antibacterial activity against multidrug-resistant strains, particularly *S. aureus* MRSA and *E. coli* ESBL, with clear inhibition zones and low MIC and MBC values. SEM analysis confirmed significant structural damage to bacterial cells, and RAPD-PCR revealed genotypic alterations compared with the control, suggesting interference with bacterial DNA. Additionally, the nanoparticles exhibited notable antioxidant and anti-inflammatory activities without cytotoxic effects on MCF-7 cells at effective concentrations. In conclusion, despite the relatively large size of the biosynthesized nanoparticles, their bactericidal effect against the studied multidrug-resistant bacteria occurred at low concentrations (1.56 µg/mL). This could be due to its phytocompound content, negative charge, or its distinctive shape. Conversely, they were nontoxic to the MCF-7 cancer cells only at concentrations above 100 µg/mL. In conclusion, these findings enhance the potential for using these green-synthesized silver nanoparticles derived from *M. parviflora* as a coating for implanted medical devices to prevent biofilm formation or in drug delivery systems, as well as a therapeutic agent against resistant pathogens. Further in vivo studies are warranted to explore their mechanisms of action and clinical applicability.

## Figures and Tables

**Table 1:** Antibacterial potential of *M. parviflora* AgNPs against some pathogenic bacteria.

Bacteria	Gram-Positive Bacteria		Gram-Negative Bacteria	
	*S. aureus* MRSA	*S. aureus* ATCC29213	*E. coli* ESBL	*E. coli* ATCC25922
	Zone of Inhibition (mm) ± Standard Deviation			
*M. parviflora* AgNPs	20.33 ± 0.88	16.67 ± 0.33	13.33 ± 0.33	16.67 ± 1.33
Gentamycin	14 ± 0	15 ± 0	14 ± 0	15.33 ± 0.33
Significance (*p* ≤ 0.01)	0.12	0.020	0.001	0.010

**Table 2:** Minimal inhibitory concentration (MIC) µg/mL and minimal bactericidal concentration (MBC) µg/mL of *M. parviflora* AgNPs.

Test Bacteria	MIC µg/mL	MBC µg/mL
*S. aureus MRSA*	0.8	1.56
*S. aureus* ATCC29213	3.125	6.25
*E. coli ESBL*	0.8	1.56
*E. coli* ATCC25922	0.8	1.56

**Table 3:** Polymorphic bands and percentage of polymorphism of each primer in *S. aureus* (ST and MRSA) treated with AgNPs from *M. parviflora* fruit extract.

Strain	*S. aureus* MRSA					*S. aureus* ATCC29213				
Primer Name	Total Bands *	Number of Bands		Percentage of Bands		Total Bands	Number of Bands		Percentage of Bands	
		Monomorphic	Polymorphic	Monomorphic	Polymorphic		Monomorphic	Polymorphic	Monomorphic	Polymorphic
Primer G02	6	4	2	67%	33%	7	2	5	29%	71%
Primer G04	3	0	3	0%	100%	10	4	6	40%	60%
Total	9	4	5	-	-	17	6	11	-	-

Total number of bands, * including bands from the control and the treatment strains.

**Table 4:** Primer sequences for RAPD-PCR.

RAPD Primer	Primer Sequence 5′-3′
G02	GGCACTGAGG
G04	AGCGTGTCTG

## Data Availability

The datasets in this research can be found in online repositories. The names of the repositories and accession numbers are included in this article.

## Abbreviations

The following abbreviations are used in this manuscript:

AgNPs	Silver nanoparticles
SEM	Scanning electron microscopy
EDX	Energy-dispersive X-ray spectroscopy
FTIR	Fourier-transform infrared spectroscopy
MIC	Minimum inhibitory concentration
MBC	Minimum bactericidal concentration
RAPD-PCR	Random amplification of polymorphic DNA–polymerase chain reaction
